# Supplementary material for: MALDI-TOF MS Profiling and Its Contribution to Mosquito-Borne Diseases: A Systematic Review
Source: Insects. 2024 Aug 29;15(9):651. doi: 10.3390/insects15090651 (PMC11432722; doi:10.3390/insects15090651)
Supplement: Supplementary file 1 [file insects-15-00651-s001.zip › insects-3160215-supplementary.pdf]

Additional Table S1. Links of articles' records from search of the databases.

Databases (extraction date)	Search terms	Link of search*
**Pubmed (17.02.23)**	Maldi AND Mosquito	https://pubmed.ncbi.nlm.nih.gov/?term=Maldi+AND+Mosquito&filter=dates.2003%2F1%2F1-2023%2F2%2F17&filter=lang.english&filter=other.excludepreprints&size=200
**Web of Science (17.02.23)**	Maldi AND Mosquito	https://www.webofscience.com/wos/woscc/summary/f5303ea2-2b86-4515-b37b-81b67d97ee85-715e2eab/relevance/1
**Science Direct (16.02.23)**	**1. (Maldi OR "mass spectrometry") AND (Mosquito) AND (surveillance OR monitoring OR management)**	
	Maldi AND "mosquito surveillance"	https://www.sciencedirect.com/search?qs=Maldi%20AND%20%22mosquito%20surveillance%22&date=2003-2023&lastSelectedFacet=articleTypes&articleTypes=REV
	Maldi AND "mosquito monitoring"	https://www.sciencedirect.com/search?qs=Maldi%20AND%20%22mosquito%20monitoring%22&years=2016&lastSelectedFacet=years
	"mass spectrometry" AND "mosquito surveillance"	https://www.sciencedirect.com/search?qs=%22mass%20spectrometry%22%20AND%20%22mosquito%20surveillance%22&date=2003-2023&articleTypes=REV%2CFLA&lastSelectedFacet=articleTypes
	"mass spectrometry" AND "mosquito monitoring"	https://www.sciencedirect.com/search?qs=%22mass%20spectrometry%22%20AND%20%22mosquito%20monitoring%22&date=2003-2023&lastSelectedFacet=articleTypes&articleTypes=FLA
	"mass spectrometry" AND "mosquito management"	https://www.sciencedirect.com/search?qs=%22mass%20spectrometry%22%20AND%20%22mosquito%20management%22&date=2003-2023&articleTypes=FLA%2CREV&lastSelectedFacet=articleTypes
	**2. (Maldi OR "mass spectrometry") AND (Mosquito) AND ("Mosquito Identification")**	
	Maldi AND "mosquito identification"	https://www.sciencedirect.com/search?qs=Maldi%20AND%20%22mosquito%20identification%22&years=2022&lastSelectedFacet=years
	"mass spectrometry" AND "mosquito identification"	https://www.sciencedirect.com/search?qs=%22mass%20spectrometry%22%20AND%20%22mosquito%20identification%22
	**3. (Maldi OR "mass spectrometry") AND (Mosquito) AND (Longevity OR Age OR oviposition)**	
	Maldi AND "mosquito longevity"	https://www.sciencedirect.com/search?qs=Maldi%20AND%20%22mosquito%20longevity%22
	Maldi AND "mosquito age"	https://www.sciencedirect.com/search?qs=Maldi%20AND%20%22mosquito%20age%22&date=2003-2023&articleTypes=REV%2CFLA&lastSelectedFacet=articleTypes
	Maldi AND "mosquito oviposition"	https://www.sciencedirect.com/search?qs=Maldi%20AND%20%22mosquito%20oviposition%22

"mass spectrometry" AND "mosquito longevity"	https://www.sciencedirect.com/search?qs=%22mass%20spectrometry%22%20AND%20%22mosquito%20longevity%22&date=2003-2023&articleTypes=FLA&lastSelectedFacet=articleTypes
"mass spectrometry" AND "mosquito age"	https://www.sciencedirect.com/search?qs=%22mass%20spectrometry%22%20AND%20%22mosquito%20age%22&date=2003-2023&articleTypes=REV%2CFLA&lastSelectedFacet=articleTypes
"mass spectrometry" AND "mosquito oviposition"	https://www.sciencedirect.com/search?qs=%22mass%20spectrometry%22%20AND%20%22mosquito%20oviposition%22&date=2003-2023&articleTypes=REV%2CFLA&lastSelectedFacet=articleTypes

4. (Maldi OR "mass spectrometry") AND (Mosquito) AND (Feed OR Blood OR engorged or "Feed behavior")

Maldi AND "mosquito feed"	https://www.sciencedirect.com/search?qs=Maldi%20AND%20%22mosquito%20feed%22&date=2003-2023&articleTypes=FLA&lastSelectedFacet=articleTypes
Maldi AND "mosquito blood"	https://www.sciencedirect.com/search?qs=Maldi%20AND%20%22mosquito%20blood%22&date=2003-2023&articleTypes=REV%2CFLA&lastSelectedFacet=articleTypes
Maldi AND "mosquito engorged"	https://www.sciencedirect.com/search?qs=Maldi%20AND%20%22mosquito%20engorged%22
Maldi AND "mosquito feed behavior"	https://www.sciencedirect.com/search?qs=Maldi%20AND%20%22mosquito%20feed%20behavior%22
"mass spectrometry" AND "mosquito feed"	https://www.sciencedirect.com/search?qs=%22mass%20spectrometry%22%20AND%20%22mosquito%20feed%22&date=2003-2023&articleTypes=REV%2CFLA&lastSelectedFacet=articleTypes
"mass spectrometry" AND "mosquito blood"	https://www.sciencedirect.com/search?qs=%22mass%20spectrometry%22%20AND%20%22mosquito%20blood%22&date=2003-2023&articleTypes=REV%2CFLA&lastSelectedFacet=articleTypes
"mass spectrometry" AND "mosquito engorged"	https://www.sciencedirect.com/search?qs=%22mass%20spectrometry%22%20AND%20%22mosquito%20engorged%22
"mass spectrometry" AND "mosquito feed behavior"	https://www.sciencedirect.com/search?qs=%22mass%20spectrometry%22%20AND%20%22mosquito%20feed%20behavior%22

5. (Maldi OR "mass spectrometry") AND (Mosquito) AND (origin OR geographic)

Maldi AND "mosquito origin"	https://www.sciencedirect.com/search?qs=Maldi%20AND%20%22mosquito%20origin%22&date=2003-2023
Maldi AND "mosquito geographic"	https://www.sciencedirect.com/search?qs=Maldi%20AND%20%22mosquito%20geographic%22
"mass spectrometry" AND "mosquito origin"	https://www.sciencedirect.com/search?qs=%22mass%20spectrometry%22%20AND%20%22mosquito%20origin%22&years=2008%2C2009%2C2020&lastSelectedFacet=years

"mass spectrometry" AND "mosquito geographic"	https://www.sciencedirect.com/search?qs=%22mass%20spectrometry%22%20AND%20%22mosquito%20%20geographic%22

6. (Maldi OR "mass spectrometry") AND (Mosquito) AND ("Mosquito disease" OR "Mosquito-borne-disease" OR MBD OR Parasite OR Arbovirus)

Maldi AND "Mosquito disease"	https://www.sciencedirect.com/search?qs=Maldi%20AND%20%22Mosquito%20disease%22&articleTypes=REV&lastSelectedFacet=articleTypes
Maldi AND "Mosquito-borne-disease"	https://www.sciencedirect.com/search?qs=Maldi%20AND%20%22Mosquito-borne-disease%22&date=2003-2023&articleTypes=REV%2CFLA&lastSelectedFacet=articleTypes
Maldi AND "Mosquito Parasite"	https://www.sciencedirect.com/search?qs=Maldi%20AND%20%22Mosquito%20Parasite%22&date=2003-2023&articleTypes=REV%2CFLA&lastSelectedFacet=articleTypes
Maldi AND "Mosquito Arbovirus"	https://www.sciencedirect.com/search?qs=Maldi%20AND%20%22Mosquito%20Arbovirus%22
"mass spectrometry" AND "Mosquito disease"	https://www.sciencedirect.com/search?qs=%22mass%20spectrometry%22%20AND%20%22Mosquito%20disease%22&date=2003-2023&articleTypes=REV%2CFLA&lastSelectedFacet=articleTypes
"mass spectrometry" AND "Mosquito-borne-disease"	https://www.sciencedirect.com/search?qs=%22mass%20spectrometry%22%20AND%20%22Mosquito-borne-disease%22&date=2003-2023&articleTypes=REV%2CFLA&lastSelectedFacet=articleTypes
"mass spectrometry" AND "Mosquito Parasite"	https://www.sciencedirect.com/search?date=2003-2023&qs=%22mass%20spectrometry%22%20AND%20%20%22Mosquito%20Parasite%22&articleTypes=REV%2CFLA&lastSelectedFacet=articleTypes
"mass spectrometry" AND "Mosquito Arbovirus"	https://www.sciencedirect.com/search?qs=%22mass%20spectrometry%22%20AND%20%22Mosquito%20Arbovirus%22

7. (Maldi OR "mass spectrometry") AND (Mosquito) AND (Bacteria OR Wolbachia)

Maldi AND "Mosquito Bacteria "	https://www.sciencedirect.com/search?date=2003-2013&qs=Maldi%20AND%20%22Mosquito%20Bacteria%22&articleTypes=FLA&lastSelectedFacet=articleTypes
Maldi AND "Mosquito Wolbachia"	https://www.sciencedirect.com/search?qs=Maldi%20AND%20%22Mosquito%20Wolbachia%22
"mass spectrometry" AND "Mosquito Wolbachia"	https://www.sciencedirect.com/search?date=2003-2023&qs=%22mass%20spectrometry%22%20AND%20%22Mosquito%20Wolbachia%22
"mass spectrometry" AND "Mosquito Bacteria "	https://www.sciencedirect.com/search?qs=%22mass%20spectrometry%22%20AND%20%22Mosquito%20Bacteria%20%22

8. (Maldi OR "mass spectrometry") AND (Mosquito) AND (“Insecticide resistance" OR "Insecticide susceptibility")

Maldi AND "Mosquito" AND “Insecticide resistance"	https://www.sciencedirect.com/search?qs=Maldi%20AND%20%22Mosquito%22%20AND%20%E2%80%9CInsecticide%20resistance%22&date=2003-

		2023&articleTypes=REV%2CFLA&lastSelectedFacet=articleTypes
	Maldi AND "Mosquito" AND “Insecticide susceptibility"	https://www.sciencedirect.com/search?qs=Maldi%20AND%20%20%22Mosquito%22%20AND%20%E2%80%9CInsecticide%20susceptibility%22&date=2003-2023&articleTypes=FLA&lastSelectedFacet=articleTypes

*Search results after selection of filters (*i.e*. year frame, type of article and language).
